# Prognostic Implications of Initial Radiological Findings of Pulmonary Fibrosis in Patients with Acute SARS-CoV-2 Infection: A Prospective Multicentric Study

**DOI:** 10.3390/diseases12110285

**Published:** 2024-11-08

**Authors:** Roxana-Elena Cîrjaliu, Sri Vidhya Gurrala, Balaji Nallapati, Vamsi Krishna, Cristian Oancea, Emanuela Tudorache, Monica Marc, Felix Bratosin, Iulia Bogdan, Ovidiu Rosca, Paula Irina Barata, Laurentiu Tony Hangan, Sergiu Ioachim Chirilă, Ariadna-Petronela Fildan

**Affiliations:** 1 Faculty of Medicine, “Ovidius” University of Constanta, 900470 Constanta, Romania; roxana.cirjaliu@365.univ-ovidius.ro (R.-E.C.); tony@medcom.ro (L.T.H.); sergiu.chirila@univ-ovidius.ro (S.I.C.); petronela.fildan@365.univ-ovidius.ro (A.-P.F.); 2 NRI Academy of Medical Sciences, NTR Health University, Mangalagiri Road, Chinakakani, Guntur, Andhra Pradesh 522503, India; srividhyagurrala@gmail.com; 3 Department of General Medicine, Katuri Medical College and Hospital, Katuri City 522019, India; dr.balajinallapati@gmail.com; 4 Sri Devaraj Urs Medical College, Kolar 563101, India; vamsikr2345@gmail.com; 5 Center for Research and Innovation in Precision Medicine of Respiratory Diseases, “Victor Babes” University of Medicine and Pharmacy Timisoara, Eftimie Murgu Square 2, 300041 Timisoara, Romania; oancea@umft.ro (C.O.); marc.monica@umft.ro (M.M.); barata.paula@student.uvvg.ro (P.I.B.); 6 Discipline of Infectious Diseases, “Victor Babes” University of Medicine and Pharmacy Timisoara, Eftimie Murgu Square 2, 300041 Timisoara, Romania; felix.bratosin@umft.ro (F.B.); iulia-georgiana.bogdan@umft.ro (I.B.); ovidiu.rosca@umft.ro (O.R.)

**Keywords:** COVID-19, pulmonary fibrosis, SARS-CoV-2, coronavirus

## Abstract

Pulmonary fibrosis detected during the acute phase of SARS-CoV-2 infection may significantly influence patient prognosis. This study aimed to evaluate the prognostic value of initial high-resolution computed tomography (HRCT) findings of pulmonary fibrosis in hospitalized COVID-19 patients and to examine how these findings relate to disease severity and clinical outcomes, with a particular focus on the development and validation of predictive scoring systems. In this multicentric prospective cohort study from January 2023 to January 2024, 120 adult patients with confirmed SARS-CoV-2 infection requiring hospitalization were enrolled from two Romanian university hospitals. Patients were categorized based on the presence (n = 60) or absence (n = 60) of pulmonary fibrosis signs on admission HRCT scans, identified by reticular opacities, traction bronchiectasis, honeycombing, and architectural distortion. Biochemical analyses, severity scores (SOFA, APACHE II, NEWS 2), and novel compound scores combining clinical and radiological data were assessed. Patients with HRCT evidence of pulmonary fibrosis had significantly higher severity scores and worse clinical outcomes. The HRCT score alone was a strong predictor of severe COVID-19 (area under the ROC curve [AUC] = 0.885), with a best cutoff value of 9.72, yielding 85.7% sensitivity and 79.8% specificity. Compound Score 1, integrating SOFA, APACHE II, and HRCT scores, demonstrated excellent predictive performance with an AUC of 0.947, sensitivity of 92.5%, and specificity of 88.9%. Compound Score 2, combining systemic inflammation markers (SIRI, SII) and NEWS 2, also showed a strong predictive capability (AUC = 0.913), with 89.2% sensitivity and 85.7% specificity at the optimal cutoff. Regression analysis revealed that Compound Score 1 had the highest hazard ratio for severe COVID-19 outcomes (HR = 4.89; 95% CI: 3.40–7.05), indicating its superior prognostic value over individual markers and traditional severity scores. Initial HRCT findings of pulmonary fibrosis are significantly associated with increased disease severity in hospitalized COVID-19 patients. The HRCT score is a valuable prognostic tool, and, when combined with clinical severity scores into Compound Score 1, it enhances the prediction of severe COVID-19 outcomes with high sensitivity and specificity. These compound scores facilitate the early identification of high-risk patients, guiding clinical decision-making and optimizing patient management to improve outcomes.

## 1. Introduction

Pulmonary fibrosis is increasingly recognized as a critical pathological feature in patients with severe acute respiratory syndrome coronavirus 2 (SARS-CoV-2), manifesting in the form of irreversible scarring and a significant decline in pulmonary function [1,2,3,4]. Radiological findings, particularly from high-resolution computed tomography (HRCT), have been pivotal in identifying fibrotic changes in COVID-19 patients, correlating these changes with disease severity, and predicting clinical outcomes. Recent guidelines, including those from the World Health Organization (WHO) and the Centers for Disease Control and Prevention (CDC), underscore the importance of imaging in managing and monitoring the progression of COVID-19, given its potential to inform therapeutic strategies and prognostication [5,6,7].

The advent of COVID-19 has seen a rapid evolution in the understanding of its pathophysiology, with a significant focus on pulmonary complications. Studies have documented a wide range of radiologic abnormalities in affected patients, with patterns resembling acute respiratory distress syndrome (ARDS) and other forms of interstitial lung disease [8,9,10]. Fibrotic changes, particularly in those progressing from acute to chronic stages of the disease, have been associated with worse outcomes, including prolonged hospital stays, increased need for mechanical ventilation, and higher mortality rates. Data from multiple international cohorts have suggested that early identification of fibrosis could be critical in tailoring treatment approaches and improving patient outcomes [11,12].

Moreover, the literature indicates a variable prevalence of fibrotic changes among patients with COVID-19, dependent on factors such as age, comorbidities, and the severity of initial illness [13,14,15]. Guidelines emphasize the role of sequential imaging to monitor disease progression and resolution, particularly in patients showing initial signs of pulmonary fibrosis. The identification of early fibrotic changes is challenging yet essential, as these patients may benefit from a different clinical management strategy, including the early initiation of anti-fibrotic therapies, which have shown promise in other fibrotic pulmonary diseases [16,17].

Despite these insights, there remains a gap in the literature regarding the specific prognostic implications of radiological findings of pulmonary fibrosis at the time of hospital admission for patients with acute SARS-CoV-2 infection. Most studies have focused on the end-stage or follow-up periods with long-term results, with fewer insights available into the acute phase implications of these findings [18,19]. Understanding the early radiological features that predict the development of fibrosis and their association with disease outcomes could guide more targeted and timely interventions, potentially improving the prognosis of these patients.

Given the current understanding and the gaps identified in the existing research, this study aims to elucidate the prognostic implications of initial radiological findings of pulmonary fibrosis for the development of severe COVID-19 in patients admitted with acute SARS-CoV-2 infection. By focusing on the acute phase of the illness, this study seeks to determine whether early fibrotic changes, as identified by HRCT at the time of admission, are predictive of COVID-19 severity. The hypothesis of this study is that early radiological signs of pulmonary fibrosis are associated with increased disease severity in patients with acute SARS-CoV-2 infection. Additionally, this study aims to establish a prognostic model that could assist clinicians in predicting the course of the disease for patients with COVID-19.

## 2. Materials and Methods

### 2.1. Study Design

This multicentric prospective cohort study was conducted in collaboration between University Ovidius from Constanța and Victor Babeș University of Medicine and Pharmacy from Timișoara. The study period extended from January 2023 to January 2024, involving patients admitted to the hospitals affiliated with these universities, both of which are equipped with advanced radiology and infectious disease units. The focus was on investigating the prognostic value of initial radiological findings of pulmonary fibrosis in patients with acute SARS-CoV-2 infection, specifically examining how these findings relate to disease severity and patient outcomes during hospitalization.

Ethical approval for this study was secured from the Institutional Review Boards of both participating universities. This approval adhered to the ethical standards of the 1964 Helsinki Declaration and its later amendments, which govern human research ethics. Informed consent was obtained from all participants or their legal guardians, ensuring that they were fully informed about the nature of this study, the confidentiality of their data, and their voluntary participation without any impact on their standard care. This work was supported by the COVIL project in the framework of the Ovidius University of Constanta biomedical grant competition, approval number 15137/05.10.2022.

### 2.2. Inclusion and Exclusion Criteria

For the study group, we selected participants based on the presence of radiological signs of pulmonary fibrosis on HRCT at admission in previously healthy individuals. For inclusion, patients were required to have a confirmed diagnosis of SARS-CoV-2, evidenced by a positive Polymerase Chain Reaction (PCR) test. All included patients were those requiring hospitalization. This study was restricted to adult patients aged 18 and above. Consent was a critical inclusion criterion, with all participants providing informed consent, affirming their understanding of and voluntary participation in this study. Only patients admitted during the designated study period from January 2023 to January 2024 were included.

The exclusion criteria were designed to minimize confounding variables and ensure the clarity of this study’s outcomes. Patients who had been hospitalized for severe COVID-19 prior to the study period were excluded to prevent prior interventions from affecting current health outcomes. Those diagnosed with co-infections, such as influenza or bacterial pneumonia at the time of admission, were also excluded to isolate the effects of COVID-19 on pulmonary fibrosis. Patients lacking complete initial HRCT scans necessary for assessing pulmonary fibrosis were excluded, ensuring the integrity of the prognostic data. Additionally, any patient who withdrew their consent was removed from this study to comply with ethical standards. Participants involved in other interventional studies that could interfere with the outcomes of interest were excluded to avoid overlapping effects. Finally, patients with severe pre-existing chronic pulmonary diseases or previous evidence of pulmonary fibrosis, which could confound the assessment of acute fibrotic changes due to COVID-19, were not included in this study.

In this prospective study, patients were carefully matched based on several critical factors to ensure comparability and control for confounding variables. Matching criteria included age, sex, and the severity of initial COVID-19 symptoms as documented at the time of admission. This approach facilitated a more precise assessment of the impact of early radiological signs of pulmonary fibrosis on patient outcomes, thereby enhancing the reliability of this study’s findings.

### 2.3. Biochemical Analysis

A comprehensive blood analysis was performed using a Sysmex XN-550 automated hematology analyzer by Sysmex Corporation, based in Kobe, Japan. This analysis included a complete blood count (CBC) and white blood cell (WBC) count, requiring 1 mL of venous blood drawn into tubes containing Ethylenediaminetetraacetic acid (EDTA) to prevent clotting and ensure the preservation of the blood sample. C-Reactive Protein (CRP) levels were determined using either a Cobas Integra 400 Plus or a Cobas e411 analyzer by Roche Diagnostics GmbH, located in Mannheim, Germany. These tests used 2 mL of blood collected in separator gel tubes designed to aid in serum separation. Additionally, interleukins were measured using enzyme-linked immunosorbent assay (ELISA) methods. Various other biochemical markers that indicate organ function and cellular damage were assessed using spectrophotometry-based biochemical analyzers.

### 2.4. COVID-19 Severity

The classification of COVID-19 severity in this study adhered to established guidelines. Patients were categorized as having either moderate or severe COVID-19 [20]. Moderate cases were those exhibiting pneumonia without severe respiratory distress symptoms. Severe cases were defined by several factors: an oxygen saturation below 93% in room air at sea level, a respiratory rate exceeding 30 breaths per minute, substantial lung infiltrates over 50% within 24 to 48 h, or clinical signs of pneumonia such as a high respiratory rate or increased oxygen needs. Critical conditions were identified by the need for mechanical ventilation, the presence of septic shock, or other significant organ dysfunction requiring intensive care.

### 2.5. HRCT Imaging

HRCT scans were performed on all patients upon admission using standardized imaging protocols. Scans were acquired with patients in the supine position during full inspiration, utilizing thin-section imaging with a slice thickness of 1–1.5 mm to ensure optimal spatial resolution. The HRCT images were independently reviewed by two experienced radiologists who were blinded to the clinical outcomes to minimize interpretation bias. The CT scores were manually estimated by the two radiologists. The criteria for identifying pulmonary fibrosis on HRCT included the presence of reticular opacities, traction bronchiectasis, honeycombing, and architectural distortion, in accordance with established radiological guidelines and the recommendations of the Fleischner Society. Recognizing that these radiological findings during the acute phase of SARS-CoV-2 infection may not represent established fibrosis but could serve as early indicators or predictors of subsequent fibrotic development, we aimed to investigate their prognostic significance.

To quantify the extent of pulmonary fibrosis on HRCT scans, we used a standardized scoring system in which the lungs were divided into five lobes: right upper, right middle, right lower, left upper, and left lower. A score from 0 to 5 was assigned to each lobe according to the percentage of fibrotic involvement: 0 (no involvement), 1 (<5%), 2 (5–25%), 3 (26–49%), 4 (50–75%), and 5 (>75%). The total HRCT score was calculated by summing the scores of all five lobes, yielding a range from 0 to 25, with higher scores indicating greater fibrotic involvement.

### 2.6. Definitions

In developing our predictive models for severe COVID-19 outcomes, we utilized several clinical scores and indices, which are abbreviated as follows: the Sequential Organ Failure Assessment (SOFA) score evaluates the extent of a patient’s organ dysfunction across multiple systems; the Acute Physiology and Chronic Health Evaluation II (APACHE II) score assesses disease severity based on physiological measurements and health history; the Computed Tomography (CT) score quantifies lung involvement observed in imaging studies. Additionally, the Systemic Inflammation Response Index (SIRI) and the Systemic Immune-Inflammation Index (SII) are biomarkers that reflect the body’s inflammatory and immune responses, respectively. The National Early Warning Score 2 (NEWS 2) is a tool used to quickly identify patients at risk of clinical deterioration by assessing vital signs. By integrating these components—SOFA, APACHE II, and CT scores in Compound Score 1, and SIRI, SII, and NEWS 2 in Compound Score 2—we aimed to enhance the prediction of severe disease progression by capturing both the clinical severity and the underlying physiological instability in COVID-19 patients.

We devised two compound scores to enhance the prediction of severe COVID-19 outcomes based on a range of clinical parameters. Compound Score 1 was calculated as a weighted average of the SOFA and APACHE II scores, with an additional component from the computed tomography score that assesses lung involvement. The formula for Compound Score 1 is defined as 2 × SOFA + 1.5 × APACHE II + 0.5 × CT Score)/4.

Compound Score 2 integrates the systemic inflammation response by combining the SIRI and the SII with the NEWS 2, which is typically used for providing early warnings of patient deterioration. The formula for Compound Score 2 is as follows: (1 × SIRI + 1 × SII + 2 × NEWS 2)/4. This score aimed to provide an early indication of severe disease progression by emphasizing systemic inflammation and physiological instability in COVID-19 patients.

### 2.7. Statistical Analysis

Statistical analyses were performed using Python version 3.8.5, utilizing libraries such as Pandas for data manipulation, SciPy for statistical tests, and Scikit-learn for logistic regression and ROC curve analysis. Descriptive statistics were employed to summarize the demographic and clinical characteristics of the patients. A Kolmogorov–Smirnov test was used to determine the normality distribution of data. Normally distributed data were represented as mean and standard deviation. Non-normally distributed data were represented by median and IQR and compared using the Mann–Whitney test. Comparative analyses between groups were conducted using the Chi-squared test for categorical variables and the *t*-test for continuous variables. Logistic regression models were developed to identify factors significantly associated with severe outcomes, quantifying these relationships through odds ratios with 95% confidence intervals. The performance of each model in predicting patient outcomes was evaluated using the area under the receiver operating characteristic curve (AUROC), with values closer to 1 indicating better predictive accuracy. The significance level was set at *p* < 0.05 for all tests.

## 3. Results

In the current study, a total of 120 COVID-19 patients were included and analyzed. Specifically, the mean age for patients with fibrosis was 58.0 ± 13.2 years compared to 55.4 ± 15.8 years for those without fibrosis (*p* = 0.326). Distribution across age categories and other demographic variables also did not differ significantly, suggesting that these factors alone did not predispose patients to the development of fibrosis post-COVID-19 infection. However, statistically significant differences were observed in the days of hospitalization and oxygen saturation levels between the two groups. Patients with evidence of pulmonary fibrosis had a longer hospital stay, averaging 13.9 ± 6.6 days, compared to 9.0 ± 7.5 days for those without fibrosis (*p* < 0.001). Additionally, lower oxygen saturation levels were noted in the fibrosis group (91.6 ± 2.7) versus the no-fibrosis group (94.2 ± 3.4) with a *p*-value of less than 0.001 (Table 1).

The laboratory findings at admission revealed significant discrepancies between COVID-19 patients with and without pulmonary fibrosis, indicative of more severe systemic involvement in the fibrosis group. Notably, all measured parameters except lymphocyte counts showed statistically significant differences (*p* < 0.001). Patients with fibrosis exhibited elevated levels of white blood cells, neutrophils, platelets, erythrocyte sedimentation rate, fibrinogen, C-reactive protein, lactate dehydrogenase, aspartate aminotransferase, alanine aminotransferase, urea, creatinine, blood glucose, and D-dimers, all exceeding the upper limits of their normal ranges. Conversely, hemoglobin levels were significantly lower in the fibrosis group (10.8 ± 1.3 g/dL) compared to the no-fibrosis group (13.5 ± 1.6 g/dL, *p* < 0.001), indicating a potential for anemia or chronic disease. The only parameter that did not differ significantly between groups was lymphocyte count (*p* = 0.899), as presented in Table 2.

The computed tomography scores, which provide a quantitative measure of lung involvement, were significantly higher in the fibrosis group (12.4 ± 4.3) compared to the no-fibrosis group (7.9 ± 3.2). Similarly, systemic inflammation and immune response markers such as the SIRI and the SII were considerably elevated in the fibrosis group, reflecting heightened inflammatory activity, similarly with the PNI, which was lower in the fibrosis group (38.5 ± 6.9).

Moreover, the severity of the clinical status as assessed by intensive care scores like the SOFA and the APACHE II were significantly higher in patients with fibrosis. The SOFA score averaged 7.3 ± 2.7 for the fibrosis group versus 3.9 ± 2.1 for the no-fibrosis group, and the APACHE II score was 17.8 ± 5.3 compared to 10.3 ± 4.0, both indicating a more critical condition among fibrosis patients (*p* < 0.001). The NEWS 2, used to detect deterioration in patients, similarly showed a higher risk in the fibrosis group (6.5 ± 2.0) than in the non-fibrosis group (3.1 ± 1.5), as seen in Table 3.

Table 4 of this study presents the best cutoff values for predicting severe COVID-19, incorporating both single markers and composite scores, with their corresponding sensitivity, specificity, and area under the curve. The HRCT score, with a cutoff of 9.7, showed high diagnostic performance with a sensitivity of 85.7%, a specificity of 79.8%, and an AUC of 0.885, indicating strong discriminative power for identifying severe cases of COVID-19 (*p* < 0.001). Other significant individual parameters included the SOFA and the APACHE II scores, which exhibited even higher sensitivity and specificity values, with SOFA achieving an AUC of 0.907 and APACHE II reaching an AUC of 0.926, both with *p*-values below 0.001.

Additionally, this study introduced two composite scores—Compound Score 1 and Compound Score 2—with cutoffs of 25.5 and 23.1, respectively. These scores synthesized multiple diagnostic indicators to enhance prediction accuracy, where Compound Score 1 achieved the highest sensitivity of 92.5% and specificity of 88.9%, reflected in an AUC of 0.947 (*p* < 0.001). Compound Score 2 also demonstrated a robust predictive capability, with a sensitivity of 89.2%, specificity of 85.7%, and an AUC of 0.913 (*p* < 0.001).

**Figure 1:**
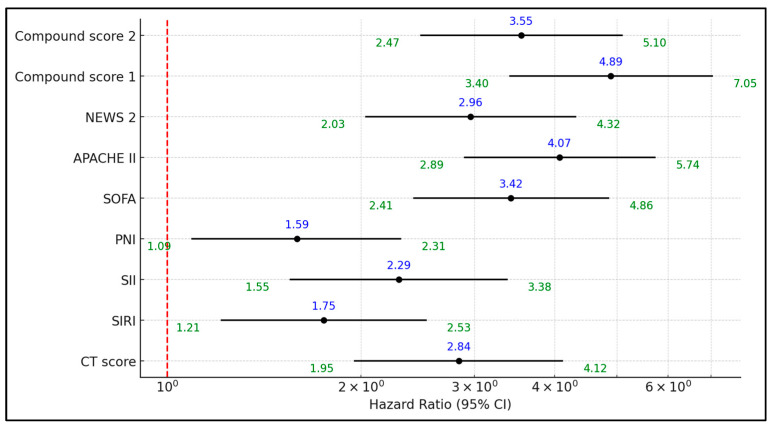
Forest plot analysis of risk for severe COVID-19 development in patients with evidence of pulmonary fibrosis at admission.

The regression analysis in Table 5 explores the hazard ratios for the development of severe COVID-19, focusing on various inflammatory markers and severity scores. The APACHE II score presented the highest hazard ratio of 4.07, significantly associating it with a greater risk of severe COVID-19 outcomes (95% CI: 2.89 to 5.74; *p* < 0.001). This was closely followed by the Compound Score 1, with a hazard ratio of 4.89, underscoring its strong predictive capability in severe disease risk stratification (95% CI: 3.40 to 7.05; *p* < 0.001). Other scores, such as the SOFA and the NEWS 2, also showed substantial risk implications with hazard ratios of 3.42 and 2.96, respectively, as presented in Figure 1.

## 4. Discussion

### 4.1. Literature Findings

The analysis revealed that elevated inflammatory markers (SIRI, SII) are closely linked to severe COVID-19 outcomes, especially in patients with fibrosis, indicating that aggressive inflammation contributes to fibrosis development. A lower PNI in this group highlights the significant impact of nutritional status on patient outcomes, underscoring the need for treatments addressing both immune response and nutrition. Higher severity scores (SOFA, APACHE II) in the fibrosis group validate their use in the early identification of high-risk patients. Compound Scores 1 and 2 combine multiple diagnostic indicators, enhance risk stratification, and guide therapeutic interventions, improving patient management and prognoses.

In a similar manner, the study by Hyun Lee et al. [21] found a pronounced susceptibility and severity of COVID-19 among patients with interstitial lung disease (ILD). Their nationwide cohort analysis in Korea demonstrated that the presence of ILD was associated with a significantly higher risk of severe COVID-19 outcomes. Specifically, they reported that 47.8% of COVID-19 patients with ILD experienced severe disease compared to only 12.6% of those without ILD, with mortality rates at 13.4% versus 2.8%, respectively (all *p* < 0.001). This stark difference highlights the profound impact of pre-existing ILD on COVID-19 severity, mirrored by the findings of Namrata Kewalramani et al. [22], who discussed the burden of post-COVID interstitial lung disease. Their review suggests a potential progression to chronic lung conditions in patients post-COVID, noting that, while some fibrotic changes resolved at 12 months, others persisted, indicating a possible evolution into chronic ILD.

Similarly, the study by Toru Arai et al. [23] explored the impact of a steroid-based treatment strategy in patients with pre-existing interstitial lung disease (preILD) and COVID-19, revealing some noteworthy outcomes. Among the small cohort of seven patients, all of whom were men with a median age of 63, those treated with corticosteroids and remdesivir showed a 30-day mortality rate of 0%, with a total mortality rate of 28.5% during the observation period. While three patients with severe COVID-19 required advanced respiratory support, two were weaned off successfully, though one eventually succumbed. This contrasts yet complements findings from Bo-Guen Kim et al. [24], who utilized a larger nationwide dataset to assess the risk of developing ILD post-COVID-19 infection and the protective effect of vaccination. They found a significantly higher risk of newly diagnosed ILD in the COVID-19 cohort compared to the controls, with an incidence rate ratio of 11.01. However, they also observed that vaccination substantially reduced this risk, with an adjusted hazard ratio of 0.44.

The study by Konlawij Trongtrakul et al. [25] demonstrated the enhanced utility of the National Early Warning Score 2 (NEWS2) when augmented with age and body mass index to predict severe COVID-19 pneumonia in a cohort of 725 patients, of whom 350 (48.3%) suffered from severe forms of the disease. Their findings showed that the NEWS2 Plus (NEWS2 with additional age and BMI parameters) significantly improved the predictive accuracy with a C-statistic of 0.821 compared to 0.798 for NEWS2 alone, validating its use in early hospital triage with a high sensitivity of 99.7% and a negative predictive value of 98.9% at a cut-off point of five. Similarly, Lauren J Scott et al. [26] explored the prognostic value of NEWS2 and its components in a multicenter study involving 1263 COVID-19 inpatients. They found that higher initial and maximum NEWS2 scores correlated with increased mortality, ICU admission, and prolonged hospital stays. Although individual respiratory parameters like oxygen saturation and respiratory rate showed predictive value for short-term mortality, with AUCs around 0.65, the composite NEWS2 score was more effective.

Other studies also evaluated various COVID-19 severity and ICU mortality scoring systems, finding that the SOFA, APACHE II, SAPS II, and 4C scores were all significant predictors of mortality, with the SOFA score showing the highest AUC [27]. They assessed 292 critically ill patients, reporting a high mortality rate of 78% and indicating that, while these scores were effective in mortality prediction, they did not significantly predict ICU or hospital length of stay. Similarly, Tom Schoenmakers [28] and colleagues examined the utility of the CoLab score within the ICU setting, observing a consistent decrease in the score over time, which correlated with improvements in patient status, such as a reduced need for mechanical ventilation and lower multi-organ failure rates. Their findings, based on linear mixed-effects models, highlight the dynamic nature of the CoLab score, which decreased by 0.30 points per day, reflecting a potential clinical tool to guide decisions about the duration of isolation and intensive care interventions.

In a similar manner, the study by Klaudia Bartoszewicz et al. [29] aimed to identify prognostic factors influencing mortality among critically ill COVID-19 patients in an ICU setting, analyzing 201 patients with a notable mortality rate of 40%. They employed logistic regression to assess various factors and found that lower interleukin 6 (Il-6) and white blood cell levels within the first 48 h of ICU admission were significant indicators of improved survival outcomes. This contrasts with the study by Juntong Wei et al. [30], which explored the biochemical underpinnings of COVID-19 severity through a lipidomic analysis of plasma samples from patients with varying disease severities. Their findings emphasized the alteration in phospholipid metabolism, particularly the ratios of specific phosphatidylcholines and lysophosphatidylethanolamines, which were significantly correlated with disease severity. Wei et al. demonstrated that lower ratios of certain phospholipids were indicative of severe COVID-19, suggesting their potential utility in early disease severity classification.

In our study, HRCT was the main tool used in classifying patients. Similarly, in other studies, HRCT was crucial in identifying the characteristic features of COVID-19 pneumonia, assessing disease severity, monitoring changes over time until resolution, and providing prognostic information to guide treatment and decisions regarding intensive care unit admission [31,32,33]. Another investigation involving 47 suspected SARS patients with normal chest radiographs found that HRCT detected atypical pneumonia in 93% of cases; all these patients experienced progressive clinical deterioration, and those with multifocal or bilateral lung involvement were more likely to require intensive care [32]. Additionally, a study of 29 SARS patients revealed that all had abnormal HRCT findings at presentation—despite normal radiographs in some—and follow-up scans showed disease progression, with a subset developing signs consistent with fibrosis, such as reticulation and mild traction bronchiectasis [33]. These findings emphasize the role of HRCT as a supplementary diagnostic tool alongside real-time polymerase chain reaction testing, enabling early detection of parenchymal changes and aiding in the management of patients with SARS-CoV-2 and SARS-associated coronavirus infections.

The clinical utility of these findings lies in their potential to significantly enhance the early identification and management of severe COVID-19 cases. By integrating initial HRCT scores and inflammatory markers into routine assessment protocols, healthcare providers can more accurately predict which patients are at higher risk for adverse outcomes, enabling the prioritization of resources such as intensive care and mechanical ventilation. Additionally, the establishment of specific cutoff values for severity scores facilitates a more stratified approach to treatment, allowing clinicians to tailor interventions that are aligned with the predicted disease trajectory. This could lead to more efficient use of healthcare resources and potentially improve survival rates by ensuring that high-risk patients receive appropriate and timely medical attention.

### 4.2. Limitations

While this study provides valuable insights into the prognostic implications of pulmonary fibrosis in COVID-19 patients, several limitations should be acknowledged. Firstly, this study’s focus on only hospitalized patients may introduce selection bias, as it excludes those with milder symptoms who manage their illness at home, potentially skewing the severity and generalizability of the findings. Additionally, the reliance on initial HRCT scans at admission could overlook the dynamic nature of pulmonary fibrosis, which may evolve significantly throughout the course of the illness. This study’s multicentric nature, though a strength in terms of diverse data, might also introduce variability in imaging techniques and clinical assessments across different centers. Lastly, the exclusion criteria, while necessary to minimize confounding factors, may limit the applicability of the results to all COVID-19 patients, particularly those with coexisting conditions that could influence both the presentation of fibrosis and the disease outcome. Further external validation is required by future studies.

## 5. Conclusions

This study demonstrates that initial HRCT findings of pulmonary fibrosis in patients with acute SARS-CoV-2 infection are significantly associated with increased disease severity and poorer clinical outcomes. Patients exhibiting fibrotic changes had longer hospital stays, lower oxygen saturation levels, and elevated inflammatory markers such as WBC, CRP, and D-dimers. Severity scores, including SOFA, APACHE II, and NEWS 2, were markedly higher in the fibrosis group, indicating a more critical clinical status. The HRCT score and the devised compound scores showed strong predictive value for severe COVID-19 outcomes, with high sensitivity and specificity. These findings suggest that the early identification of pulmonary fibrosis through HRCT can serve as a valuable prognostic tool, aiding clinicians in risk stratification and guiding therapeutic interventions to improve patient management and outcomes.

## Figures and Tables

**Table 1:** Background characteristics and demographics of COVID-19 admitted patients with and without evidence of pulmonary fibrosis.

Variables	Fibrosis (n = 60)	No Fibrosis (n = 60)	*p*-Value
Age (mean ± SD)	58.0 ± 13.2	55.4 ± 15.8	0.326
Age category			0.319
<40 years	5 (8.3%)	8 (13.3%)	
40–59 years	39 (65.00%)	31 (51.7%)	
≥60 years	16 (26.7%)	21 (35.00%)	
Gender			0.353
Men	33 (54.6%)	38 (63.3%)	
Women	27 (45.4%)	22 (36.7%)	
Place of origin			0.274
Rural	11 (18.2%)	16 (26.7%)	
Urban	49 (81.8%)	44 (73.3%)	
COVID-19 vaccination			0.458
Yes	27 (45.5%)	23(38.3%)	
No	33 (54.5%)	37 (61.7%)	
Smoking			0.425
No	44 (72.7%)	40 (66.7%)	
Past smoker	16 (27.37%)	20 (33.3%)	
Days since symptom onset (median, IQR)	2.7 (1.1–4.5)	2.8 (1.3–4.9)	0.518
Days of hospitalization (mean ± SD)	13.9 ± 6.6	9.0 ± 7.5	<0.001
Oxygen saturation (mean ± SD)	91.6 ± 2.7	94.2 ± 3.4	<0.001
Developed severe disease	27 (45.0%)	13 (21.7%)	0.006

SD—Standard Deviation.

**Table 2:** Common laboratory parameters measured at admission in COVID-19 patients with and without evidence of pulmonary fibrosis.

Variables	Normal Range	Fibrosis (n = 60)	No Fibrosis (n = 60)	*p*-Value
WBC (×10^3^/L)	4.0–10.0	16.7 ± 4.6	12.3 ± 5.9	<0.001
Hemoglobin (g/dL)	12.0–16.0	10.8 ± 1.3	13.5 ± 1.6	<0.001
Neutrophils (×10^3^/L)	2.0–7.0	12.4 ± 3.1	9.4 ± 4.9	0.001
Lymphocytes (×10^3^/L)	1.0–3.0	3.9 ± 1.4	3.9 ± 2.7	0.899
Platelets (×10^3^/uL)	150–400	328.4 ± 68.2	274.3 ± 55.9	<0.001
ESR (mm/h)	<20	47.9 ± 9.2	19.6 ± 5.9	<0.001
Fibrinogen (mg/dL)	200–400	602.9 ± 98.8	348.2 ± 72.1	<0.001
CRP (mg/L)	<5	120.8 ± 32.2	22.5 ± 9.6	<0.001
LDH	100–250	420.4 ± 60.2	234.7 ± 45.3	<0.001
AST (U/L)	0–40	84.3 ± 22.5	28.8 ± 10.6	<0.001
ALT (U/L)	0–40	68.6 ± 21.1	26.8 ± 9.3	<0.001
Urea (mg/dL)	15–45	59.2 ± 15.3	35.9 ± 10.5	<0.001
Creatinine (mg/dL)	0.6–1.2	1.4 ± 0.3	0.9 ± 0.2	<0.001
Blood glucose (mg/dL)	70–140	182.3 ± 40.6	118.3 ± 29.8	<0.001
D-dimers (ug/mL)	0.0–0.5	3.6 ± 1.0	0.6 ± 0.2	<0.001

AST—Aspartate Aminotransferase; ALT—Alanine Aminotransferase; CRP—C-reactive Protein; WBC—White Blood Cells; LDH—Lactate Dehydrogenase.

**Table 3:** Comparison of severity scores among COVID-19 patients with and without evidence of pulmonary fibrosis.

Variables	Fibrosis (n = 60)	No Fibrosis (n = 60)	*p*-Value
HRCT score	12.4 ± 4.3	7.9 ± 3.2	<0.001
SIRI	2.9 ± 1.1	1.2 ± 0.6	<0.001
SII	950.3 ± 310.4	460.2 ± 289.8	<0.001
PNI	38.5 ± 6.9	48.3 ± 5.4	<0.001
SOFA	7.3 ± 2.7	3.9 ± 2.1	<0.001
APACHE II	17.84± 5.3	10.3 ± 4.0	<0.001
NEWS 2	6.5 ± 2.0	3.1 ± 1.5	<0.001

HRCT—High-Resolution Computed Tomography; SIRI—Systemic Inflammation Response Index; SII—Systemic Immune-Inflammation Index; PNI—Prognostic Nutritional Index; APACHE—Acute Physiology and Chronic Health Evaluation; NEWS—National Early Warning Score.

**Table 4:** Best cutoff values for severe COVID-19 prediction.

Laboratory Parameter	Best Cutoff Value	Sensitivity	Specificity	AUC	*p*-Value
HRCT score	9.7	0.857	0.798	0.885	<0.001
SIRI	2.0	0.763	0.749	0.819	0.006
SII	675.3	0.824	0.766	0.854	<0.001
PNI	42.6	0.701	0.684	0.739	0.033
SOFA	6.1	0.877	0.831	0.907	<0.001
APACHE II	16.5	0.903	0.865	0.926	<0.001
NEWS 2	5.4	0.836	0.793	0.874	0.001
Compound Score 1	25.5	0.925	0.889	0.947	<0.001
Compound Score 2	23.1	0.892	0.857	0.913	<0.001

HRCT—High-Resolution Computed Tomography; SIRI—Systemic Inflammation Response Index; SII—Systemic Immune-Inflammation Index; PNI—Prognostic Nutritional Index; APACHE—Acute Physiology and Chronic Health Evaluation; NEWS—National Early Warning Score.

**Table 5:** Regression analysis for severe COVID-19 development based on the inflammatory markers.

Factors Above the Best Cutoff	Hazard Ratio	95% CI Lower	95% CI Upper	*p*-Value
HRCT score	2.84	1.95	4.12	0.001
SIRI	1.75	1.21	2.53	0.003
SII	2.29	1.55	3.38	<0.001
PNI	1.59	1.09	2.31	0.016
SOFA	3.42	2.41	4.86	<0.001
APACHE II	4.07	2.89	5.74	<0.001
NEWS 2	2.96	2.03	4.32	<0.001
Compound Score 1	4.89	3.4	7.05	<0.001
Compound Score 2	3.55	2.47	5.1	<0.001

HRCT—High-Resolution Computed Tomography; SIRI—Systemic Inflammation Response Index; SII—Systemic Immune-Inflammation Index; PNI—Prognostic Nutritional Index; APACHE—Acute Physiology and Chronic Health Evaluation; NEWS—National Early Warning Score.

## Data Availability

Data available upon request from the authors.
